# IL12/23 Blockade with Ustekinumab as a Treatment for Immune-Related Cutaneous Adverse Events

**DOI:** 10.3390/ph16111548

**Published:** 2023-11-02

**Authors:** Stephanie L. Gu, Tara Maier, Andrea P. Moy, Stephen Dusza, David M. Faleck, Neil J. Shah, Mario E. Lacouture

**Affiliations:** 1 Dermatology Service, Department of Medicine, Memorial Sloan Kettering Cancer Center, New York, NY 10021, USA; gus@mskcc.org (S.L.G.);; 2 Department of Dermatology, Weill Cornell Medical College, New York, NY 10021, USA; 3 Department of Pathology and Laboratory Medicine, Memorial Sloan Kettering Cancer Center, New York, NY 10065, USA; 4 Gastroenterology, Hepatology, and Nutrition Service, Memorial Sloan Kettering Cancer Center, New York, NY 10065, USA; 5 Department of Medicine, Weill Cornell Medical College, New York, NY 10021, USA; 6 Genitourinary Oncology Service, Department of Medicine, Memorial Sloan Kettering Cancer Center, New York, NY 10065, USA

**Keywords:** immune-related cutaneous adverse events, ustekinumab, biologics, dermatologic adverse events, immune-related adverse events

## Abstract

**Background**: Immune-related cutaneous adverse events (ircAEs) are frequent and may reduce quality of life and consistent dosing. IL12/23 has been implicated in psoriasis, which is reminiscent of the psoriasiform/lichenoid ircAE phenotype. We report the use of ustekinumab as a therapeutic option. **Methods**: Patients at Memorial Sloan Kettering Cancer Center, New York, who received immune checkpoint inhibitors and were treated with ustekinumab or had the keywords “ustekinumab” or “Stelara” in their clinical notes between 1 March 2017 and 1 December 2022 were retrospectively identified via a database query. Documentation from initial and follow-up visits was manually reviewed, and response to ustekinumab was categorized into complete cutaneous response (CcR, decrease to CTCAE grade 0), partial cutaneous response (PcR, any decrease in CTCAE grade exclusive of decrease to grade 0), and no cutaneous response (NcR, no change in CTCAE grade or worsening). Labs including complete blood count (CBC), cytokine panels, and IgE were obtained in a subset of patients as standard of care. Skin biopsies were reviewed by a dermatopathologist. **Results**: Fourteen patients with psoriasiform (85.7%), maculopapular (7.1%), and pyoderma gangrenosum (7.1%) ircAEs were identified. Ten (71.4%) receiving ustekinumab had a positive response to treatment. Among these 10 responders, 4 (40%) demonstrated partial cutaneous response and 6 (60%) demonstrated complete cutaneous resolution. Six patients (42.9%) experienced interruptions to their checkpoint inhibitor treatment as a result of intolerable ircAEs, and following ircAE management with ustekinumab, two (33.3%) were successfully rechallenged with their checkpoint inhibitors. On histopathology, patients primarily had findings of interface or psoriasiform dermatitis. No patients reported an adverse event related to ustekinumab. **Conclusions**: Ustekinumab showed a benefit in a subset of patients with psoriasiform/lichenoid ircAEs. No safety signals were identified. However, further prospective randomized controlled trials are needed to confirm our findings.

## 1. Introduction

The recent advent of immunotherapies as validated treatment options has redefined the cancer therapy landscape [1,2,3,4,5]. Despite the efficacy, up to 90–100% of all patients treated with anti-cytotoxic T-lymphocyte-associated protein-4 (anti-CTLA-4) inhibitors, anti-programmed cell death-1 (PD-1)/programmed cell death ligand-1 (PD-(L)1) inhibitors, or combination immunotherapies develop treatment-related adverse events of a variety of grades [6,7]. Immune-related cutaneous adverse events (ircAEs) are among the most common immune-related adverse events (irAEs) and develop the earliest, highlighting the importance of their detection [8,9,10].

These ircAEs are most often managed with topical steroids and/or oral antipruritics, including antihistamines and gabapentinoids. Refractory cases may require treatment with systemic steroids. However, this is undesirable given the number of complications associated with these medications and requires caution in patients on active treatment for cancer given their possible influence on anti-tumor efficacy. In recent years, biologics for ircAE management have emerged as a safe and efficacious alternative to steroid-based regimens.

Ustekinumab (UST), an IL-12 and IL-23 inhibitor, is FDA approved for the treatment of moderate to severe psoriasis and inflammatory bowel disease [11,12,13]. IL-12/23 may influence the development of ircAEs, but their exact role is unclear. Previous studies have reported on the successful use of UST as an off-label treatment for immune-related colitis (ircolitis) and ircAEs, but the existing literature is sparse. In this retrospective single-center study, we evaluate the efficacy and safety of UST as a treatment for ircAEs.

## 2. Results

### 2.1. Demographics and Oncologic History

A total of 14 patients (median age 71 (28–95), 42.9% female) with advanced cancers undergoing treatment with immune checkpoint inhibitors were included in the study (Table 1). The most common cancers included genitourinary malignancies (*n* = 7, 50%), melanoma (*n* = 4, 28.6%), pancreatic cancer (*n* = 1, 7.1%), breast cancer (*n* = 1, 7.1%), and lymphoma (*n* = 1, 7.1%). Patients were most often on anti-PD1/PDL1 monotherapy with pembrolizumab (*n* = 9, 64.3%), nivolumab (*n* = 3, 21.4%), avelumab (*n* = 1, 7.1%), or atezolizumab (*n* = 1, 7.1%). Median time to ircAE development was 102 days (9–911).

### 2.2. Clinicopathologic Characteristics

**Figure 1:**
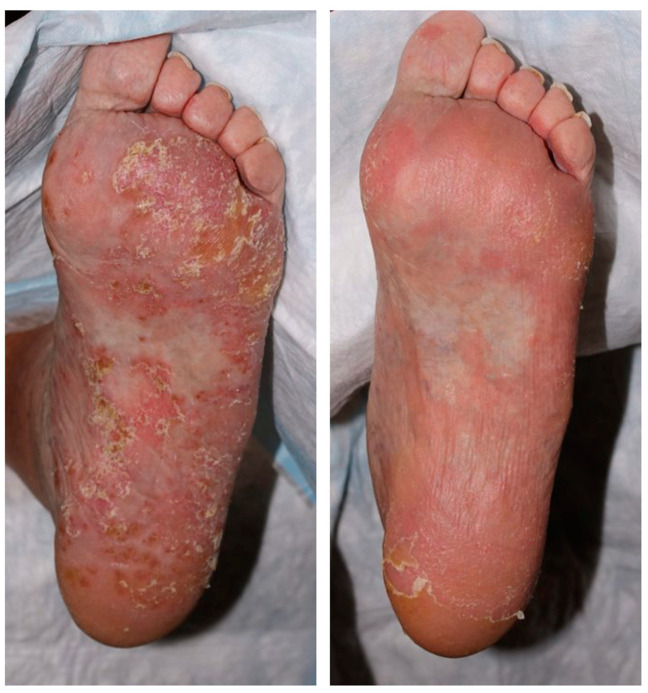
Representative clinical image of patient with palmoplantar psoriasis who received treatment with UST.

The ircAE phenotypes included psoriasiform eruptions (*n* = 12, 85.7%), pyoderma gangrenosum (*n* = 1, 7.1%), and morbilliform eruption (*n* = 1, 7.1%). The median interval between the start of ircAE and initiation of UST was 47.5 days (5–1327) (Figure 1). Eleven patients received a skin biopsy before the initiation of UST. Histopathologic review of skin biopsies most often showed interface dermatitis (*n* = 7) (with scattered neutrophils or eosinophils noted in two cases each); one of these cases showed a dense lichenoid lymphocytic infiltrate with eosinophils and a subepidermal split. Psoriasiform dermatitis with intracorneal neutrophils was seen in four cases (one of which showed concomitant interface changes). One case showed a dense dermal and perifollicular mixed inflammatory infiltrate with focal abscess formation compatible with early pyoderma gangrenosum (Table 2).

### 2.3. Clinical Response to UST

**Figure 2:**
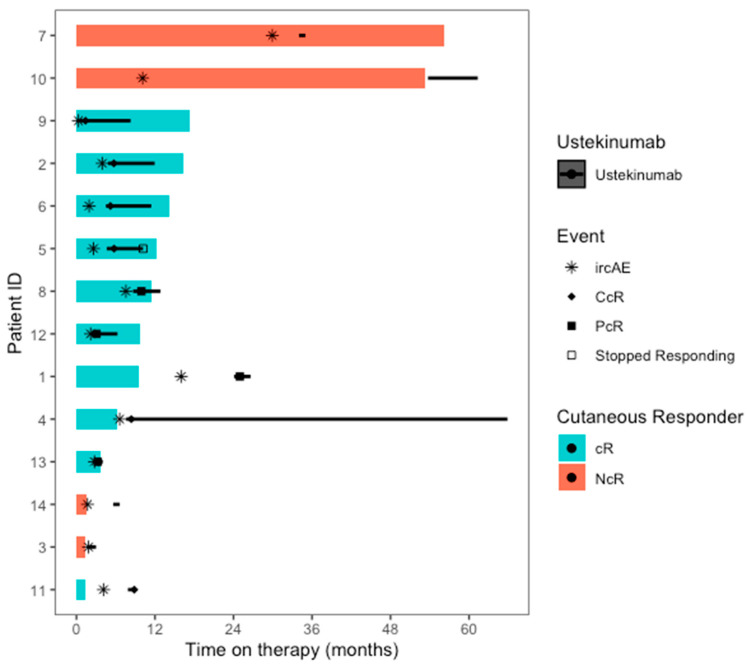
Swimmer’s plot of the time in therapy for both ICB therapy (colored bars) and ustekinumab (black bars), along with the timing of specific events within the treatment the course (*n* = 14). NcR= cutaneous non-responder to ustekinumab, cR = cutaneous responder to ustekinumab, IrcAE = immune-related cutaneous adverse event, CcR = complete cutaneous response to ustekinumab, PcR = partial cutaneous response to ustekinumab.

Ten patients (71.4%) responded to UST. Of these, four (40%) had a PcR response and six (60%) had a CcR response (Figure 2). There was no statistically significant difference in age, sex, race, ethnicity, or cancer type between responders and non-responders. Response was observed in most patients (*n* = 9, 75%) with psoriasiform ircAEs and in the patient with pyoderma gangrenosum. No response was observed in the patient with morbilliform ircAE. The median duration of treatment was 123 days (1–1774), and the median time from UST start to documented response was 27 days (21–34) in patients with CcR and 24 days (7–39) in patients with PcR. The median total doses of UST received per patient was 7.5 (2–9) and 3 (2–4) for patients with CcR and PcR, respectively. 

Seven patients experienced an interruption of cancer therapy as a result of ircAEs. Two patients responded to UST and were successfully rechallenged with their respective regimens. The remaining five patients were not rechallenged due to progression of the disease (*n* = 1), no evidence of disease at the time of ICI discontinuation (*n* = 1), or the severity of irAEs (ircAEs *n* = 2; irAEs *n* = 1). 

### 2.4. Changes in Supportive Management of ircAEs

**Figure 3:**
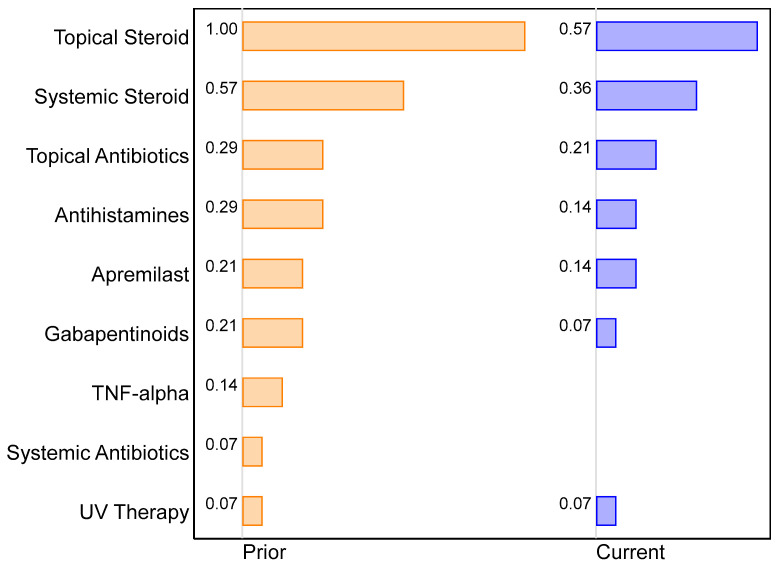
Two-way bar chart of the proportion of patients on specific treatments prior to and concurrent with ustekinumab (*n* = 14).

The median number of supportive treatments decreased from three to two following initiation of UST (Figure 3). Prior to treatment with UST, most patients had been treated with topical steroids (*n* = 14, 100%), systemic steroids (*n* = 8, 57.1%), antihistamines (*n* = 4, 28.6%), topical antibiotics (*n* = 4, 28.6%), gabapentinoids (*n* = 3, 21.4%), apremilast (*n* = 3, 21.4%), infliximab (*n* = 2, 14.3%), systemic antibiotics (*n* = 1, 7.1%), and/or UV therapy (*n* = 1, 7.1%). Following initiation of UST, patients remained on concurrent topical steroids (*n* = 8, 57.1%), systemic steroids (*n* = 5, 35.7%), topical antibiotics (*n* = 3, 21.4%), antihistamines (*n* = 2, 14.3%), apremilast (*n* = 2, 14.3%), gabapentinoids (*n* = 1, 7.1%), and UV therapy (*n* = 1, 7.1%). In addition, one patient initiated treatment with topical calcipotriene and betamethasone combination cream alongside UST therapy (Figure 3).

### 2.5. Safety of Ustekinumab

Overall, the median number of UST injections administered was 3.5 (1–9). In accordance with the dosing recommendations for plaque psoriasis, an initial 45 or 90 mg injection was administered subcutaneously at day 0 and at 4 weeks, followed by 45/90 mg injections every 12 weeks until symptoms were managed. No patients reported any adverse events related to their UST use. 

### 2.6. Laboratory Values

Before treatment with UST, 1 out of 11 patients (9.1%) had elevated IgE, 3 out of 9 patients (33.3%) had elevated IL-5 levels, and 8 out of 10 patients (80%) had elevated IL-6 levels. There was no statistically significant difference in neutrophils, eosinophils, lymphocytes, absolute neutrophils, absolute eosinophils, absolute lymphocytes, WBCs, IgE, IL-5, or IL-6 before and after treatment with UST. 

### 2.7. Anti-Tumor Response and Cancer Progression

Of the 14 patients included in the study, 8 (57.1%) experienced progression of disease (POD) during or after treatment with UST. Six of these eight patients had metastatic disease, and four patients had progressive disease before the initiation of UST. Median time to POD from initiation of UST among these patients was 3.6 months (0.2–12.9). Four patients (57.1%) were transitioned to another cancer therapy regimen and three (42.9%) were transitioned to hospice care. Median time to next treatment (TTNT) for all patients with POD was 11.4 months (8.1–30.4) and 15.1 months (8.1–30.4) for those with POD in the setting of metastatic disease. 

Eleven patients were receiving ICI treatment for metastatic cancer, and three were receiving ICIs as adjuvant therapy. All of these patients remained in remission throughout their treatment with UST and continued to maintain this status at the most recent follow-up (median 12.7 months (9.63–58.4) from UST initiation, median 21.0 months (4.7–76.8) from ICI initiation). Of the patients with advanced disease before UST initiation, four (36.4%) had progressive disease, four (36.4%) had stable disease, and three (21.4%) had an overall response, including one (9.1%) with complete response and two (18.2%) with partial response. At three months following UST start, three patients (27.3%) demonstrated a progression of their tumor, five (45.5%) had stable disease, one (9.1%) had complete response, and two (18.2%) had partial response. At the most recent follow up (median 15.8 months (1.64–26.0) from UST start), six (54.5%) demonstrated a progression of their tumor, three (27.3%) had stable disease, one (9.1%) had complete response, and one (9.1%) had partial response. 

## 3. Discussion

### 3.1. Clinical Response to Ustekinumab

UST was an effective treatment for ircAEs in our cohort, with 71.4% responding positively. This is clinically significant, as ircAEs occur in up to 80–90% of patients and frequently cause patient morbidity [7]. UST appears to have the most clinical utility for psoriasiform eruptions, which was the primary ircAE phenotype in our cohort. In addition, this therapy is effective for steroid- or treatment-refractory ircAEs, as all patients in our study had previously failed at least one other therapy. 

Notably, one patient experienced concurrent immune-related colitis (ircolitis), which is another common cause of morbidity and mortality among patients receiving ICIs [14]. Although this patient’s colitis did not respond to UST, this treatment has been used successfully for this indication and may therefore be particularly useful in patients with concurrent ircolitis and ircAE [15]. This is significant, as using a single therapy to manage multiple toxicities can reduce patient burden and risk of treatment-related AEs. 

Although UST is safe for long-term use, treatment can be tapered once symptoms are adequately managed. In addition, patients may experience resolution of their ircAEs following completion of their ICI therapy, and attempts to discontinue UST at that time are reasonable. However, ircAEs may persist for months to years following cessation of ICI therapy, and any changes in treatment should be made at the discretion of the managing clinician.

Conventional therapies for the management of ircAEs include topical and systemic steroids and oral antipruritics, including antihistamines and gabapentinoids. Although effective, these therapies are associated with numerous adverse events (AEs). Gastrointestinal, metabolic, and psychologic toxicities frequently complicate long-term treatment with systemic steroids [16,17,18]. In addition, systemic steroid treatment prior to or concurrent with ICI treatment can inhibit anti-tumor efficacy and reduce patient survival [19,20,21,22,23]. Topical steroids have also been associated with numerous cutaneous toxicities [24].

Antihistamines and gabapentinoids are frequently used as an adjunct to topical therapies but may be sedating and should be used with caution in elderly patients. In addition, gabapentinoids may be abused, especially among high-risk patients with a history of substance use or other psychiatric disorders [25]. Given the frequency and possible severity of the AEs associated with conventional ircAE therapies, treatment with UST, which has a favorable long-term safety profile, should be considered.

### 3.2. Mechanism of Ustekinumab for ircAE Treatment

Blockade of IL12 and 23 with UST reduced the severity of ircAEs among our patients. IL-12 and IL-23 are known to coordinate activity between the innate and adaptive immune systems, but the role of these cytokines in the development of ircAEs is unclear [26]. Patients with severe immune-related adverse events (irAEs), including ircAEs, express higher levels of IL12 than patients without severe irAEs [27]. However, some evidence demonstrates that IL-12 plays a protective role against the development of skin inflammation [28]. IL-23 appears to play an opposing role, promoting inflammation through the release of IL-17, and has been implicated in several irAEs [29,30]. Therefore, we speculate that IL12/23 blockade may exert its therapeutic effect primarily by antagonizing IL-23.

### 3.3. Safety of Ustekinumab

UST is safe for long-term usage, with adverse events occurring at rates comparable to those seen with placebo treatment [31]. Though side effects in severely ill patients (such as upper respiratory tract infections and nasopharyngitis) have been reported, serious side effects from UST are rare [11,12,32]. No patients in our cohort reported any AEs to UST.

Prior research has shown that IL12 and 23 play integral roles in cancer biology, and blockade of these with UST may influence tumor progression or response to antineoplastics [33]. Through stimulation of IFN-γ synthesis, IL12 represses tumor growth and metastasis [34,35]. IL23 appears to have the opposite effect and has been shown to increase carcinogenesis and tumor activity in animal systems exposed to exogenous IL-23 [36,37,38]. The interplay between IL12 and 23 is complex, and clinicians should be aware of this potential risk when prescribing UST to patients undergoing active treatment for cancer.

Although this understanding is critical, our cohort was unfortunately underpowered to address this question. In addition, the heterogenous tumor types of our patients make it difficult to draw any conclusions regarding the relationship between UST use and tumor progression. It is worth noting that the subset of patients who received ICI as an adjuvant therapy did not report any tumor recurrence. Although some patients receiving ICIs as primary treatment did experience progression of disease, 57.1% had ongoing tumor progression before initiation of UST and 75% received systemic steroids, which have been shown to undermine anti-tumor efficacy. In addition, two patients were able to continue ICI post UST treatment, which is encouraging.

Our study has several strengths and limitations. Despite our study involving the first and largest cohort to describe the effects of UST on ircAEs, our sample size is small. In addition, it includes multiple tumor types and ICI treatments that were used in different settings (neo/adjuvant vs. metastatic), thus making it difficult to understand the anti-tumor effects of UST. Overall, our study provides compelling evidence to further investigate UST for refractory ircAEs, especially for psoriasiform ircAEs.

## 4. Materials and Methods

### 4.1. Patients

This retrospective study was conducted at Memorial Sloan Kettering Cancer Center (MSKCC) in New York, NY, USA, with institutional review board approval under protocol #16-458. Patients undergoing active treatment with immune checkpoint inhibitors (ICIs) (ipilimumab, pembrolizumab, nivolumab, cemiplimab, atezolizumab, avelumab, or durvalumab) as either monotherapy or in combination with other agents between 1 March 2017 and 22 December 2022 who received at least one dose of UST for the treatment of ircAEs were identified. Clinical records were then manually reviewed for clinical outcome data. 

### 4.2. Study Outcomes

The primary aim of this study was to assess the clinical response to treatment of ircAEs with UST. Clinical response was evaluated within 30 days of treatment with UST. Severity of ircAEs was graded using the Common Terminology Criteria for Adverse Events version 5.0 [39]. The clinical response to ircAEs was defined as complete cutaneous response (CcR, reduction to grade 0), partial cutaneous response (PcR, any reduction in grade), or no cutaneous response (NcR, no clinical improvement or worsening of symptoms). A board-certified oncodermatologist evaluated all patients’ ircAE phenotypes. Patient clinical images were stored and extracted from an automated imaging system (Vectra^®^ by Canfield Scientific^®^ Parsippany-Troy Hills, NJ, USA) and were used to determine ircAE grade or response when not documented in the clinical record.

The secondary aims of this analysis included assessment of AEs related to UST and characterization of laboratory and histopathologic findings of treated ircAEs. Absolute eosinophil, lymphocyte, and neutrophil counts; percentage of eosinophils, lymphocytes, and neutrophils; white blood cell counts; and immunoglobulin E (IgE) and interleukin 5 (IL-5) levels were compared before and after treatment with UST, and all available ircAE biopsies were evaluated by a board-certified dermatopathologist. In addition, cancer status prior to the initiation of UST, three months post initiation of UST, and at the last follow-up was obtained via a review of the clinical record to assess the influence of UST treatment on tumor progression.

### 4.3. Statistical Methods

Descriptive and relative frequencies were used to describe the patient population, the characteristics of their underlying malignancy, the ircAEs experienced, and the subsequent treatment with UST. Differences between responders and non-responders were explored using Wilcoxon rank sum tests, Fisher’s exact tests, and paired t-tests. Analyses were performed with Stata v16.1 (Stata Corporation, College Station, TX, USA) and RStudio.

## 5. Conclusions

We describe the first and largest study to evaluate the safety and efficacy of UST for steroid refractory ircAEs. We observed clinically meaningful improvement in refractory ircAEs, especially psoriasiform eruptions. Our results are promising, but further randomized controlled prospective study is warranted to fully understand the safety and efficacy of UST as a treatment for ircAEs. 

## Figures and Tables

**Table 1:** Characteristics of cancer patients receiving ustekinumab for ircAEs.

		All Patients (*n* = 14)	Responders to Ustekinumab (*n* = 10)	Non-Responders to Ustekinumab (*n* = 4)	*p*-Value
**Age**	**Median (IQR)**	71 (10)	71 (24)	72 (12)	0.515
**Sex, *n* (%)**	**Female**	6 (42.9)	3 (75)	3 (30)	0.125
	**Male**	8 (57.1)	1 (25)	7 (70)	
**Race, *n* (%)**	**White**	14 (100)	4 (100)	10 (100)	--
	**Non-Hispanic**	14 (100)	4 (100)	10 (100)	--
**Cancer type, *n* (%)**	**Breast cancer**	1 (7.1)	1 (25)	0 (0)	0.708
	**Lymphoma**	1 (7.1)	0 (0)	1 (10)	
	**Melanoma**	4 (28.6)	1 (25)	3 (30)	
	**Pancreatic**	1 (7.1)	0 (0)	1 (10)	
	**Genitourinary**	7 (14.3)	1 (25)	1 (10)	
**ICB type, *n* (%)**	**Atezolizumab**	1 (7.1)	0 (0)	1 (10)	0.375
	**Avelumab**	1 (7.1)	0 (0)	1 (10)	
	**Nivolumab**	3 (21.4)	2 (50)	1 (10)	
	**Pembrolizumab**	9 (64.3)	2 (50)	7 (70)	

**Table 2:** Characteristics of ircAEs.

		All Patients (*n* = 14)	Responders to Ustekinumab (*n* = 10)	Non-Responders to Ustekinumab (*n* = 4)	*p*-Value
**IBC infusions before ircAE**	**Median (IQR)**	3.5 (4)	3 (4)	13 (27)	0.226
**Days from ICB to ircAE**	**Median (IQR)**	47.5 (112)	31.5 (70)	122 (663)	0.296
**ircAE phenotype, *n* (%)**	**Psoriasiform**	12 (85.7)	9 (90)	3 (75)	0.689
	**Morbilliform**	1 (7.1)	0 (0)	1 (25)	
	**Pyoderma gangrenosum**	1 (7.1)	1 (10)	0 (0)	
**Baseline ircAE CTCAE 5.0 grade, *n* (%)**	**1**	3 (21.4)	2 (20)	1 (25	0.869
	**2**	6 (42.9)	4 (40)	2 (50)	
	**3**	5 (35.7)	4 (40)	1 (25)	

## Data Availability

Data is contained in this article. More detailed data will be provided in response to reasonable requests.
